# Short-term blood pressure variability and outcomes in non-dialysis chronic kidney disease

**DOI:** 10.3389/fmed.2022.911205

**Published:** 2022-09-27

**Authors:** Ge Wang, Kai Ma, Zhilan Ma, Xiaoyan Guo, Yan Wang, Lan Ma, Chenchen Qi, Yan Li, Xiaoling Zhou

**Affiliations:** ^1^Department of Nephrology, The Second Affiliated Hospital of Xi'an Jiaotong University, Xi'an, China; ^2^Department of Chest Surgery, Xi'an International Medical Center Hospital, Xi'an, China; ^3^Department of Nephrology, General Hospital of Ningxia Medical University, Yinchuan, China; ^4^Department of Nephrology, NO215.Hospital of Shaanxi Nuclear Industry, Xianyang, China; ^5^Department of Nephrology, The First People's Hospital of Yinchuan, Yinchuan, China

**Keywords:** chronic kidney disease, blood pressure variability, cardiovascular disease, renal outcome, ambulatory blood pressure monitoring

## Abstract

**Background:**

Blood pressure variability (BPV) is associated with cardiovascular and all-cause mortality, and has been demonstrated in dialysis patients, but has been poorly studied and remains controversial in non-dialysis chronic kidney disease (CKD) patients. We investigated the effect of short-term BPV on prognosis in this population.

**Methods:**

A total of 245 stage 1–4 CKD patients with 24-h ambulatory blood pressure recordings were recruited. BPV was evaluated by standard deviation, coefficient of variation, and variation independent of the mean, respectively. All subjects were followed up to the composite end-point event or until January 15, 2020. Patients were divided into two groups based on 24-h median variation independent of the mean, and demographics, laboratory indicators and echocardiogram results were compared. Logistic regression was used to analyze the risk factors for increased BPV. Multivariate Cox regression and Kaplan-Meier survival analysis were used to explore the relationship between BPV and renal prognosis and major cardiovascular events.

**Results:**

The mean age was 42.07 ± 12.66 years, with 141 males (57.55%). Multivariate Logistic regression analysis showed that high BMI (OR 1.110, *P* = 0.017), hyperkalemia (OR 2.227, *P* = 0.040), increased left ventricular end-diastolic diameter (OR 1.103, *P* = 0.010) and hypertension (OR 2.525, *P* = 0.002) were independent risk factors for high BPV. Kaplan-Meier survival analysis showed that renal and cardiovascular outcomes were better in the low BPV group than in the high BPV group (*P* = 0.006; *P* = 0.002). After adjusting for age, sex and traditional kidney related risk factors, BPV were not independently associated with renal outcomes. High BPV (HR 4.662, *P* = 0.017) was the main independent risk factor for major cardiovascular events in CKD.

**Conclusions:**

In non-dialysis CKD, short-term BPV was associated with major cardiovascular disease but not renal progression. BMI, hypertension, potassium balance, and left ventricular end-diastolic diameter influenced short-term BPV.

## Introduction

Problems associated with aging are becoming more and more severe throughout the world and high blood pressure (BP) is prevalent in the elderly. Epidemiological data indicate that the global prevalence of CKD is approximately 8–16% and increasing from year to year (1). In 2010, a cross-sectional study conducted in China revealed that the prevalence of CKD in China was 10.8%, or about 119.5 million adults (2).

Poor control of hypertension can result in kidney damage or worsen the initial kidney, or cardiovascular disease. A study in China showed that the awareness rate of hypertension in CKD patients was 80.7%, the treatment rate was 95.6%, but the control rate was only 57.1% (3). BP fluctuates greatly in CKD patients and is difficult to control due to abnormal activation of the renin-angiotensinogen-aldosterone system, water and sodium retention, insulin resistance and other factors, it is often necessary to take three or more antihypertensive drugs (4, 5). As a result, stability monitoring and accurate BP assessment are essential to patients with CKD.

The fluctuation of BP over a certain period of time is called blood pressure variability (BPV), including long term (visit-to-visit), mid-term (day-by-day), short term (within 24h) and very short term (beat-by-beat) BPV. Prior studies have demonstrated that BPV independent of mean BP is associated with the occurrence of cardiovascular disease (6, 7) as well as incident stroke in people with hypertension (8). Sarafidis et al. conducted a large, cross-sectional study and found that BPV increased with decreasing estimated glomerular filtration rate (eGFR) (9). In hemodialysis patients, some studies documented that BPV was related to cardiovascular, cerebrovascular and all-cause death (10, 11). However, in CKD patients who do not yet require dialysis, the influence of BPV on renal disease and its value in determining long-term prognosis remains to be elucidated (12). For patients with stage 1–4 CKD, it is of great importance to protect residual renal function, delay the need for renal replacement therapy, reduce the occurrence of CVD, and improve the prognosis. In stage 1**-**4 CKD patients who have not yet started renal replacement therapy, does this relationship still exist? We conducted a single-center retrospective study on this issue.

## Materials and methods

### Subjects

This was a retrospective, longitudinal, observational study performed in the Department of Nephrology, General Hospital of Ningxia Medical University on all non-dialysis CKD patients who were diagnosed and followed up regularly from January 1, 2012 to December 31, 2018. The inclusion criteria were age >18 but <70, those with a follow-up time of ≥1 year, and those who completed 24-h ambulatory blood pressure monitoring (ABPM). We excluded patients who had undergone renal replacement therapy, including hemodialysis, peritoneal dialysis, kidney transplantation, or patients with a history of clearly diagnosed cardiovascular and cerebrovascular diseases, including coronary heart disease, myocardial infarction, malignant arrhythmia, cerebral infarction, cerebral hemorrhage and those with any tumor or those with missing follow-up data. All patients voluntarily participated in this study and signed an informed consent form. This project was approved by the Ethics Committee of Ningxia Medical University.

### Study design

Patients were divided into two groups based on the median 24 h systolic blood pressure VIM at baseline. (1) high BPV group (VIM >11.96) and (2) low BPV group (VIM ≤ 11.96). The demographic information collected on patients included age, gender, smoking history, BMI (weight/height^2^) and previously diagnosed chronic diseases. Laboratory tests performed on the enrolled patients included blood potassium, sodium, calcium, phosphorus, albumin, hemoglobin, 24-h urine protein quantification, uric acid, blood urea nitrogen, creatinine, and eGFR (calculated by CKD-EPI formula), The composite endpoints included the occurrence of renal progression (creatinine doubling, initiation of maintenance hemodialysis or peritoneal dialysis), major cardiovascular events (coronary heart disease, myocardial infarction, heart failure, malignant arrhythmia, cerebral infarction, and cerebral hemorrhage) and death. The study termination was January 15, 2020, or the occurrence of any of the endpoint events for individual patients. Follow-up was done on an outpatient basis and by telephone every 1–3 months.

### Short-term BPV measurement

The Welch Allyn ABPM 6,100 non-invasive ambulatory blood pressure monitor was used and the cuff was worn on the left upper arm. ABPM started at around 8.am and readings were taken every 30 min during the day and every 1 h during the night, and was stopped after 24 h on the next day. During the monitoring period, patients were told to avoid strenuous activities and emotional agitation, but otherwise maintain their normal lifestyle. We used standard deviation (SD), coefficient of variation (CV = SD / mean), and variation independent of the mean (VIM) to quantify the 24 h blood pressure variation range. The VIM was calculated according to the following formula:

VIM = k × SD/mean^X^, where ^X^ is the curve–fitting coefficient of each patient's SD (dependent variable) and *mean* is the BP mean (independent variable); and k = M^X^, where M is the mean blood pressure of all subjects (13).

### Echocardiography

Left ventricular septal thickness (LVST), left ventricular posterior wall thickness (LVPWT), left ventricular end diastolic diameter (LVDD), left ventricular ejection fraction (LVEF) and body surface area (BSA) were recorded to calculate left ventricular mass (LVM) and left ventricular mass index (LVMI).

Devereux (14) LVM(g) = 0.832 × [(LVDD+LVST+LVPWT)^3^- LVDD^3^]+0.6

LVMI (g/m) = LVW/BSA

BSA (15) = 0.0061 × height (cm) + 0.0128 × weight (kg)−0.1529

### Statistical analyses

The data are expressed as mean ± standard deviation for normal distribution, and the non-normal data are expressed as median (interquartile interval, M, as (Q1, Q3, etc.). *T*-test was used to compare the two groups with normal data, and a non-parametric test was used to compare the two groups with non-normal data. The chi-squared test was used to compare the counting data between groups. The Kaplan-Meier method was used to analyze the relationship between BPV and kidney prognosis and major cardiovascular events. We used multiple Logistic regression analysis to explore the factors of BPV. A multivariate Cox regression model was used to analyze the risk factors of renal prognosis and cardiovascular and cerebrovascular events. The difference was statistically significant at *P* < 0.05), and α = 0.05 was the test level.

## Results

This study investigated 271 patients with non-dialysis CKD who underwent 24 h ABPM. We excluded 12 patients with incomplete data, four patients with previously diagnosed cerebrovascular disease, and two patients older than 70 years. Eight patients were excluded because of lack of follow-up. Ultimately, we included 245 non-dialysis stage 1–4 CKD patients to study the relationship between short-time BPV and the outcomes. There were 141 males (57.55%) and the average age was 42.07 ± 12.66 years. There were 135 patients with hypertension (55.1%) and 31 patients with diabetes (12.65%). With respect to etiology, there were 212 patients (86.53%) with primary glomerular disease, 13 (5.31%) with diabetic nephropathy, 10 (4.08%) with hypertensive nephropathy and 10 with other diseases. Renal biopsy was performed on 185 subjects, among which 78 (42%) had IgA nephropathy, 55 (30%) had membranous nephropathy and 18 (10%) patients had glomerular microlesions as the main pathological types, while 34 patients exhibited other pathological types. Table 1 presents the baseline characteristics for all subjects, as well as differences in baseline data between the higher and lower BPV groups. The median eGFR was 81.77 mL /min/1.73m^2^, with 102 (41.63%) patients in stage CKD1, 67 (27.35%) in stage CKD2, 59 (24.08%) in stage CKD3, and 17 (6.94%) in stage CKD4.

**Table 1:** Baseline demographics, clinical characteristics, and 24-h ambulatory BP parameters, in the study cohort composed of 245 patients with CKD.

**Variable**	**Overall**	**VIM ≤ 11.96(123)**	**VIM > 11.96(122)**	***P*-value**
Age (yrs)	42.1 ± 12.7	40.27 ± 12.26	43.85 ± 12.86	0.028
Men, *n* (%)	141 (58%)	64 (52.03%)	77 (63.11%)	0.079
BMI (kg/m2)	24.7 ± 3.6	23.8 ± 3.29	25.62 ± 3.66	<0.001
Smoking history, *n* (%)	69 (28%)	39 (31.71%)	36 (29.51%)	0.641
Diabetes, *n* (%)	31 (21%)	8 (6.5%)	23 (18.85%)	0.004
Hypertension, *n* (%)	135 (55%)	49 (39.84%)	86 (70.49%)	<0.001
Potassium (mmol/l)	4.1 ± 0.4	4.06 ± 0.36	4.18 ± 0.38	0.008
Calcium (mmol/l)	2.1 ± 0.2	141.41 ± 2.35	141.27 ± 2.49	0.647
Phosphorous (mmol/l)	1.2 ± 0.2	2.14 ± 0.2	2.1 ± 0.18	0.107
Creatinine (umol/l)	91.4 (69,128.6)	89.1 (68.6,117.4)	93.5 (70.53,139.75)	0.163
Uric acid (umol/L)	370.13 ± 99.79	355.49 ± 98.95	384.9 ± 98.85	0.021
Proteinuria (g/d)	1.9 (0.7,3.8)	1.61 (0.62,3.02)	2.43 (0.84,4.16)	0.014
Blood glucose (mmol/l)	4.8 (4.44,5.32)	4.76 (4.44,5.25)	4.83 (4.41,5.35)	0.931
Hemoglobin (g/l)	138.6 ± 22.2	140.52 ± 22.39	136.7 ± 21.97	0.179
Total cholesterol (mmol/l)	5.0 (4.0,6.64)	4.82 (3.87,6.45)	5.05 (4.09,7.03)	0.413
24h SBP (mmHg)	120 (111,133.5)	114 (107,121.5)	129 (118,141)	<0.001
24h DBP (mmHg)	77 (70,84)	73 (66.5,81)	78.5 (74,86)	<0.001
24h PP (mmHg)	44 (38.5,51)	41 (37,46)	48 (41,56)	<0.001
24h SBPSD	11.81 (9.58,14.27)	9.59 (8.45,10.8)	14.27 (12.96,17.08)	<0.001
24h DBPSD	9.7 (8.2,11.46)	8.25 (7.5,9.39)	11.37 (10.04,12.84)	<0.001
24h SBPCV	9.8 (8.2,11.7)	8.23 ± 1.52	11.58 (10.24,13.34)	<0.001
24h DBPCV	12.9(11.2,15.3)	11.73 ± 2.38	14.6 (12.76,16.57)	<0.001
Morning BP surge (mmHg)	25.59 ± 14.32	20.37 ± 9.78	30.86 ± 16.17	<0.001
LVST (mm)	9 (8,9)	9 (8,9)	9 (8,9)	0.029
LVPW (mm)	9 (8,9)	9 (8,9)	9 (8,9)	0.026
LVDD (mm)	47.69 ± 3.92	46.8 ± 3.67	48.6 ± 3.97	<0.001
LVEF (%)	68.68 ± 4.98	68.9 ± 4.05	68.45 ± 5.77	0.480
LVM (g)	144.57 ± 30.88	136.8 ± 26.25	152.4 ± 33.23	<0.001
LVMI (g/m)	82.36 ± 16.64	79.45 ± 13.25	85.28 ± 19.09	0.006

**Figure 1:**
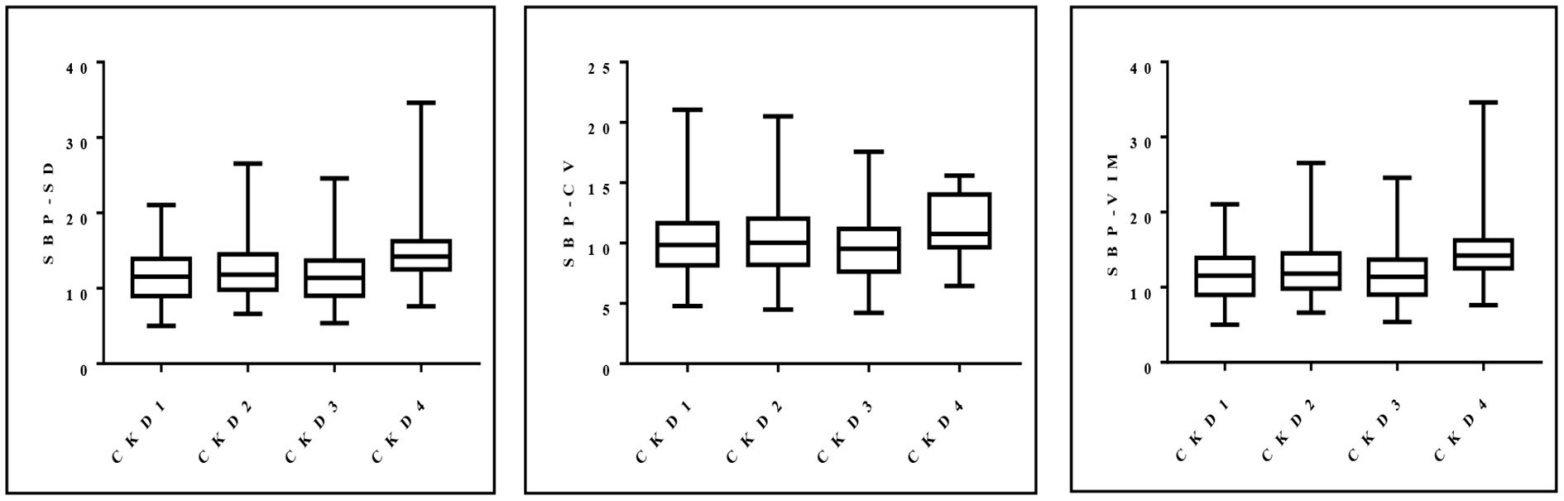
Distribution of SD, CV and VIM of systolic blood pressure in patients with stages 1–4 CKD.

Blood pressure variability (VIM = 15.03) in patients with CKD4 was significantly greater than that in patients with CKD1 (VIM = 11.89) (*P* = 0.002). This trend persisted even when SD and CV were used as quantitative indicators of blood pressure variability (*P* = 0.002; *P* = 0.032) (Figure 1).

Patients in the higher BPV group were older, had higher BMI, and higher average BP. There was no difference in the distribution of BPV between men and women or between the low and high groups (*P* = 0.079). In terms of clinical indices, the blood potassium, uric acid and urinary protein were higher in the high BPV group, but there was no significant difference in serum creatinine distribution. In addition, the patients in the higher BPV group had greater left ventricular thickness and left ventricular mass, which also meant they had larger hearts and limited diastolic and systolic function.

### Risk factors for increased BPV

We included the above variables (*P* <0.1) in the multivariate logistic regression analysis to determine the risk factors for increased BPV. High BMI (OR 1.110, *P* = 0.017), hyperkalemia (OR 2.227, *P* = 0.040), increased left ventricular end-diastolic diameter (OR 1.103, *P* = 0.010) and hypertension (OR 2.525, *P* = 0.002) were all statistically significant as risk factors for elevated BPV (Table 2).

**Table 2:** Factors that contribute to the increase of BPV.

**Variable**	***OR***	**95% *CI***	***P*–value**
BMI (kg/m^2^)	1.110	1.019–1.209	0.017
Hypertension	2.525	1.420–4.491	0.002
Potassium (mmol/l)	2.227	1.038–4.777	0.040
LVDD (mm)	1.103	1.023–1.189	0.010

### Outcomes

**Figure 2:**
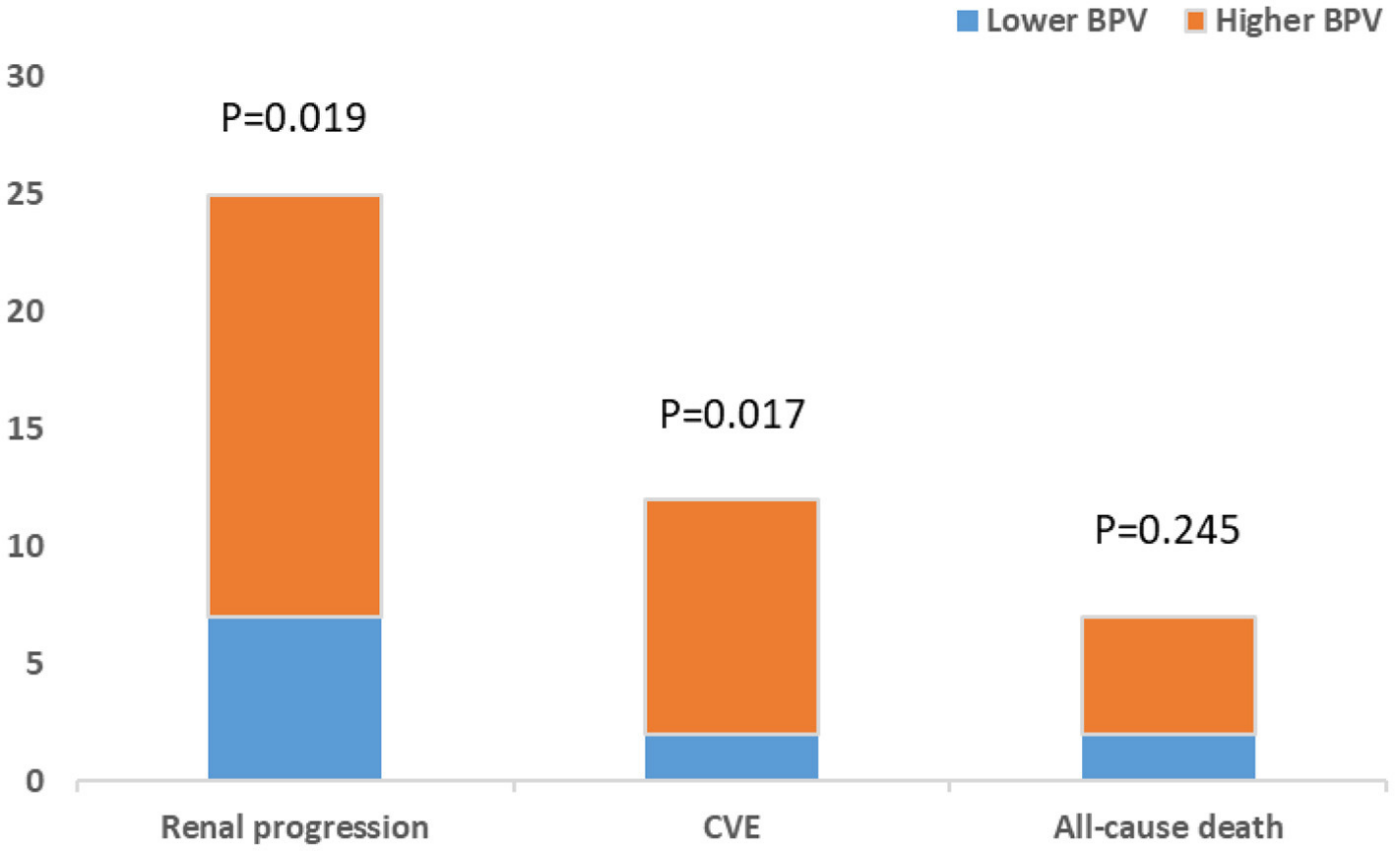
Comparison of renal progression, cardiovascular events and all-cause death between higher BPV and lower BPV group.

**Figure 3:**
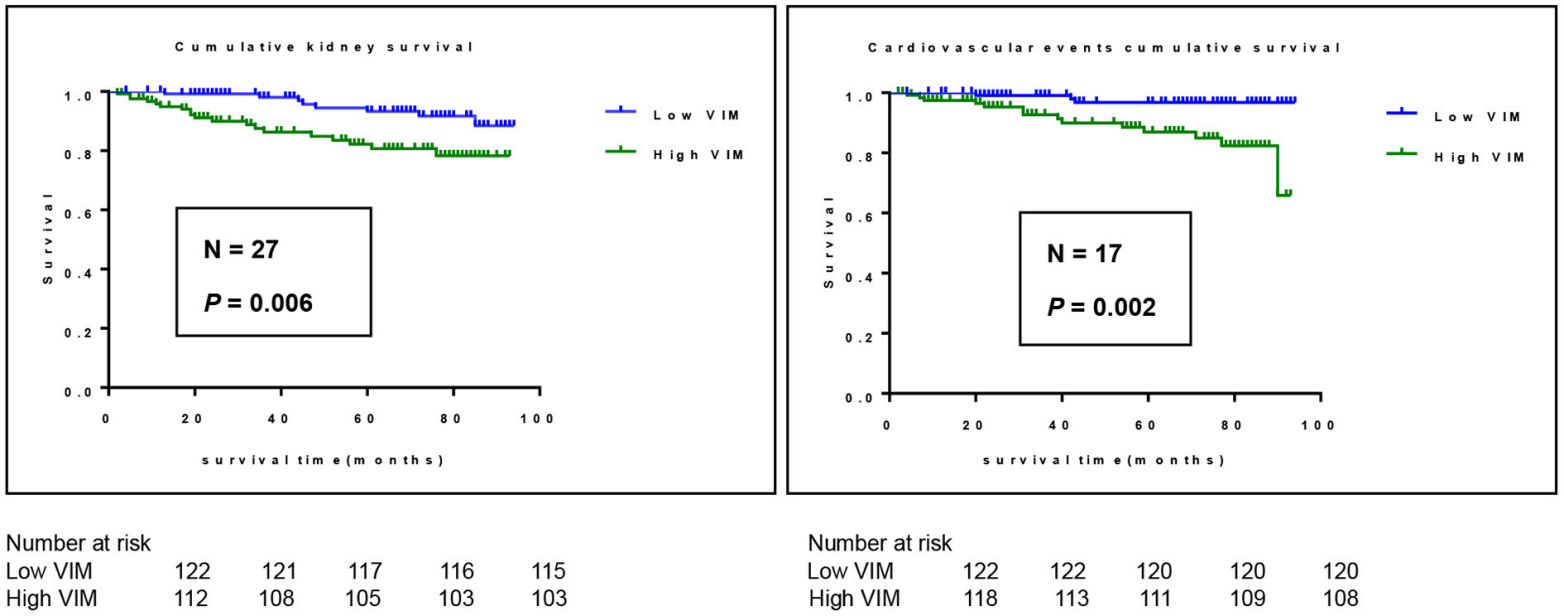
Kaplan-Meier survival curves for outcome of cardiovascular events and renal progression in participants stratified according to 24 h SBP-VIM. Cardiovascular events include all cerebrovascular and cardiovascular events.

A cohort of 245 patients with non-dialysis CKD were enrolled, with a maximum follow-up time of 94 months and a median follow-up time of 64 months. A total of 44 patients had multiple endpoints, among which 25 had renal endpoints, 12 had major cardiovascular events and 7 patients died. We investigated the effects of BPV on kidney disease progression and major cardiovascular events in patients with non-dialysis CKD patients. Figure 2 shows the renal progression, incidence of major cardiovascular events all-cause deaths, and comparison of the incidence of different end-points in each group. We observed an obvious increase in renal progression and incidence of cardiovascular events in the higher BPV group (*P* < 0.05), but there was no statistical difference in all-cause mortality (*P* > 0.05). After Kaplan-Meier survival analysis, renal prognosis in the low BPV group was significantly greater than that in the high BPV group (log rank = 7.444, *P* = 0.006). Similar results were also seen in major cardiovascular prognosis. Patients in the low BPV group had better cardiovascular outcomes than those in the high BPV group during follow-up (log rank = 10.03, *P* = 0.002) (Figure 3). The association between 24 h SBP-VIM and the risk of renal progression and major cardiovascular events was further investigated with the Cox proportional hazard model. High SBP-VIM (> VIM 11.96) was positively correlated with renal progression without adjustment (HR = 2.998, *P* = 0.009). However, the association disappeared in fully adjusted models. High SBP-VIM (> VIM 11.96) was always associated with the occurrence of cardiovascular events in both unadjusted and fully adjusted models (HR = 4.704, *P* = 0.022) (Table 3).

**Table 3:** Multivariate Cox proportional hazard model showing association of high SBP–VIM with renal progression and cardiovascular events.

**Model**	**HR**	**95% CI**	***P*–value**
**Risk of renal progression**			
Unadjusted	2.998	1.309–6.866	0.009
Adjusted for age and sex	2.886	1.246–6.683	0.013
Adjusted for age, sex, and baseline eGFR–EPI	2.67	1.128–6.321	0.025
Adjusted for age, sex, baseline eGFR–EPI, DM, SBP, and DBP	1.739	0.686–4.408	0.243
**Risk of cardiovascular disease**			
Unadjusted	5.898	1.693–20.542	0.005
Adjusted for age and sex	4.671	1.330–16.401	0.016
Adjusted for age, sex, BMI, SBP and DBP	4.663	1.241–17.520	0.023
Adjusted for age, sex, BMI, SBP, DBP and HB	4.236	1.120–16.024	0.033
Adjusted for age, sex, BMI, SBP, DBP, HB, LVM and LVMI	4.704	1.253–17.658	0.022

HB, Hemoglobin; DM, diabetes mellitus.

## Discussion

The purpose of this study was to analyze blood pressure variability in patients with non-dialysis CKD, identify the risk factors that may increase BPV, and explore the relationship between BPV and prognosis. During the follow-up of this study (longest follow-up period, 94 months; median follow-up period, 64 months), a total of 44 end-point events occurred. With the Kaplan-Meier survival analysis, we observed that the high VIM group with low VIM had worse renal prognosis (*P* = 0.006) and higher risk of major cardiovascular events in (*P* = 0.002). We also demonstrated that high SBP-VIM (> VIM 11.96) was independently associated with major cardiovascular events but not renal progression in patients with pre-dialysis CKD. Most studies have shown that long-term and short-term BPV were associated with target organ damage (such as heart, kidney, and brain) and all-cause death in the general population (16–18) and in people with hypertension (19, 20), however, some studies, like ours, have not found an association with renal prognosis after full adjustment (21–23). Therefore, we consider that BPV is strongly associated with major cardiovascular events and all-cause mortality, but further prospective studies of renal progression with larger sample sizes are needed.

We selected those factors that may contribute to increased BPV subjected them to multifactorial logistic regression analysis, and determined that high BMI, hyperkalemia, increased left ventricular end-diastolic diameter and hypertension were the main risk factors responsible for BPV increase. CKD patients with high BMI, excess blood volume, and a sedentary lifestyle with lack of exercise were prime candidates for increased blood pressure fluctuations. They also showed a tendency to abnormal lipid metabolism, often accompanied by high blood pressure and high blood sugar, in addition to insulin resistance and other factors leading to atherosclerosis and loss of vascular elasticity. Chen et al. found a positive association between BMI and average real variability (ARV) of systolic BPV (24). In a study of risk factors for BPV in hemodialysis patients, Feng at al. found that age and weight gain during hemodialysis were independent risk factors for BPV (25). Hyperactivity of sympathetic nerves, increased catecholamine concentration, activation of the RAAS system, increased cardiac afterload, progressive left ventricular hypertrophy and increased end diastolic diameter of the left ventricle in patients with CKD, is associated with the dysregulation of cardiac BP control and increased fluctuation of BP. Persistent high BP can lead to increased pressure in the glomeruli, glomerular fibrosis or atrophy, impaired regulation of body fluid balance and decreased production of active vascular compounds, and metabolic disorders, aggravating the severity of hypertension and BPV even further. When patients with CKD develop hypertension, sympathetic nerves become hyperactive, the concentration of catecholamine increases, the RAAS system is activated, cardiac afterload increases, and left ventricular hypertrophy gradually occurs, while the left ventricular end diastolic diameter increases, the regulatory effect of the heart on blood pressure is weakened, and BPV increases. Low urinary potassium excretion was independently associated with high BPV in a Korean study of 1,860 patients with pre-dialysis chronic kidney disease and a median follow-up of approximately 5.6 years (26).This may suggest that low urinary potassium excretion is an important mechanism of high BPV in CKD patients. We may be the first to find a relationship between blood potassium levels and BPV that is not yet supported by the literature. However, studies on the effects of potassium intake levels on BPV as well as the cardiovascular system suggest that a certain level of potassium intake (90–120 mmol/day) may be a protective factor for blood pressure and cardiovascular events in patients with CKD (27). However, this may also have the risk of hyperkalemia. At present, there have been few reports about the correlation between serum potassium and BPV, and further prospective studies with large samples are needed to test this hypothesis.

It is clear that the definition of BPV is not unequivocal. In the case of traditional ‘dippers' (fall in nighttime systolic and diastolic BP >10% from day-time BP), non- dippers (fall in nighttime BP <10%), inverted dippers (night-time BP fall of 10%-20%) and extreme dippers (night-time BP fall >20%) current methods can only represent the variation of BP at night. They have no capacity for quantifying the variation of BP throughout the whole 24 h or even during observation. Several studies have confirmed that the prevalence of the non-dipper BP pattern in patients with CKD is higher than that in patients with essential hypertension and is related to damage of the kidney, cardiovascular system and other target organs in patients with CKD (28–30). Researchers began to use statistical parameters such as SD, CV, weighted standard deviation (wSD), VIM and ARV to calculate BP fluctuation within a period of events. However, SD, CV and wSD were always based on average blood pressure and could not independently explain the correlation between BPV and prognosis. ARV averages the difference between successive BP readings over a specific time period and is widely used in many clinical studies of BPV, especially in long-term BPV (31). VIM is derived from more complex calculations such as curve fitting of SD and mean BP and has greater value in scientific research (32). Several studies found that when VIM was used as a measure of BPV, it was considered a better parameter than certain others because it was independent of mean BP (33). In this study, SD, CV and VIM were selected to evaluate BPV. In addition, ambulatory BP monitoring was more representative than clinical readings, and VIM was ultimately selected for grouping and as the main indicator.

It was noted that some prognostic studies also found numbers of biomarkers associated with end-stage renal disease (ESRD) or cardiovascular events in patients with CKD. Some of these biomarkers may have analyzed the etiological and predictive links demonstrated with clinical results. Cystatin C is a low molecular weight protein produced by nucleated cells that is freely filtered through the glomerulus and not secreted by renal tubules. It is completely reabsorbed. Shin et al. found that cystatin C was a better predictor of cardiovascular events and mortality than creatinine or Egfr (34). FGF-23 is a widely studied biomarker, which has been proved to be closely related to atherosclerosis, calcium and phosphorus metabolism disorder and renal function progression in patients with CKD (35). At present, some markers, such as hypersensitive troponin and NT-pro BNP, have been widely used in clinical practice, which are considered by clinicians to be closely related to acute myocardial infarction and heart failure. GDF-15, a member of the TGF-β family of cytokines, has been found to be involved in apoptosis repair and growth. GDF-15 may be a predictor of incidence of CKD, eGFR decline (36) and CVD independent of traditional CV risk factors, renal function, and other biomarkers (C-reactive protein, B-type natriuretic peptide, cardiac troponin) (37). There are still some controversial biomarkers to be further studied, which is of great significance for clinicians to improve the early detection of the prognosis and complications of CKD.

## Limitations

The limitations of this study are mainly in the following aspects. First, we are not able to clarify the casual relation between high BPV and the kidney outcome and major cardiovascular events in non-dialysis CKD patients. In this study, the patients' conditions were relatively mild at enrollment. 169(69%) patients in stage 1 and 2 CKD, and the number of endpoint events observed was small. Thus, our study may be underpowered to detect adverse kidney outcomes within the specific follow-up time. Second, single-center retrospective assessments with small sample size may have bias that is difficult to account for. Third, although we found that BPV was independently associated with several factors such as larger BMI and hyperkalemia in our study, the mechanisms between them are not particularly well defined. In particular, the pathophysiological mechanisms between blood potassium levels and BPV need to be confirmed in animal models or in randomized controlled trials. In the future, large-sample, multi-center, prospective clinical studies are needed to further explore the impact of BPV on the prognosis of CKD patients, and whether lowering BPV can delay the progression of non-dialysis CKD and improve the prognosis. Lastly, due to the limitation of time and data, we only analyzed the effect of BPV at baseline on prognosis of enrolled patients. However, over the long course of CKD, there are many factors affecting BPV that cause it to change dynamically, and how to detect and manage them will require much future effort.

## Data availability statement

The original contributions presented in the study are included in the article/supplementary material, further inquiries can be directed to the corresponding author.

## Author contributions

GW conceived, designed, and coordinated the writing of the whole manuscript. KM, XG, and YL revised literature. ZM and LM provided clinical study of this work and collected clinical data. YW and CQ made statistical analysis. XZ took part in study design and responsible for the final draft. All the authors contributed to this manuscript and approved the submitted version.

## Funding

This paper was supported by Ningxia Science Foundation (Project number: 2019A0288) and Foundation for Returned Scholars of Ningxia (Project number: 2017659).

## Conflict of interest

The authors declare that the research was conducted in the absence of any commercial or financial relationships that could be construed as a potential conflict of interest.

## Publisher's note

All claims expressed in this article are solely those of the authors and do not necessarily represent those of their affiliated organizations, or those of the publisher, the editors and the reviewers. Any product that may be evaluated in this article, or claim that may be made by its manufacturer, is not guaranteed or endorsed by the publisher.
